# “I don’t hide my feelings, even though I try to”: insight into teacher educator emotion display

**DOI:** 10.1007/s13384-013-0129-5

**Published:** 2013-10-08

**Authors:** Gerda Hagenauer, Simone E. Volet

**Affiliations:** 1 Department of Educational Research, Salzburg University, Erzabt-Klotz-Straße 1, 5020 Salzburg, Austria; 2 School of Education, Murdoch University, South Street, Murdoch, Perth, WA 6150 Australia

**Keywords:** Emotion expression, Emotion display, Emotion suppression, Emotion regulation, Teacher education, Higher education teaching

## Abstract

This article addresses the issue of teacher educators’ emotion display when teaching and interacting with students. Little is known about this phenomenon in higher education generally, and teacher education specifically. An empirical study was conducted to address this gap by investigating teacher educators’ views on appropriate and inappropriate emotion display and its functions in the process of teaching. The study also examined how teachers used emotion regulation strategies to manage the intensity of their experienced emotions. The participants (six male, nine female) were from two public Australian universities and were all teaching first-year students in pre-service education. Data were collected through in-depth, semi-structured face-to-face interviews. Qualitative analyses revealed that these teachers viewed the open expression of positive emotions as an integral aspect of their teaching practice. In terms of negative emotions, they reported the criticality of controlling such experiences, and the occasional need to completely conceal them. Some reflected on the instrumental functions and conscious use of emotion display and emotion suppression. Findings are discussed in light of prior research; limitations of this exploratory study are addressed, and directions for future research are outlined.

## Introduction

This article focuses on teacher educators’ emotion display, a dimension of emotion regulation (Gross 1998, 2002), when teaching and interacting with students. An empirical study investigated how teacher educators manage and communicate their emotions, and how possible consequences of emotions are expressed and regulated.

How teachers express or suppress their emotions is significant to education research for two main reasons. Firstly, the suppression or masking of emotion can trigger negative effects for an individual generally (e.g., an increase in experienced emotional intensity; reduced available cognitive capacity; less satisfying relationships; Butler and Gross 2004; Richards and Gross 1999; Rivers et al. 2007), and for individuals in the workplace specifically (e.g., a decrease in job satisfaction and workplace well-being, or an increase in symptoms of burn-out; Chang 2009; Lechuga 2012). Secondly, appropriate emotional display is crucial for successful teaching (e.g., unregulated anger leads to reduced concentration on the teaching process), student learning, and for establishing positive student–teacher relationships (Oplatka 2011; Sutton 2007).

If a teacher is capable of managing his/her emotions, emotion display can be applied instrumentally to achieve specific (teaching) goals (e.g., Tamir 2011), as the shown or hidden emotions serve as contextual social information for students (Keltner and Haidt 1999; Tiedens and Leach 2004). In a review of theory and research on emotional competence, Garner (2010, p. 311) observed that students in school “are highly attuned to their teachers’ emotional expressions, and teachers’ expressions of emotions can either positively influence students’ comprehension and understanding of the subject matter or detract from their learning.”

Although there is empirical evidence of the relevance of teachers’ emotion management in the school context (e.g., Oplatka 2011; Schutz and Zembylas 2009), research on teaching in higher education has largely neglected the nature and significance of teachers’ emotions (for exceptions, see for example Hagenauer and Volet 2013; Lahtinen 2008; Postareff and Lindblom-Ylänne 2011; Trigwell 2012), their emotional display in classroom situations and interaction with students, as well as their functions and consequences. The study reported in this paper addresses this gap, focusing specifically on teacher educators in higher education. Teacher educators are a special group of teachers in higher education for several reasons: First, they often have school experience themselves and therefore are likely to be more attuned to the “ethical and humanistic dimensions underlying teaching occupation” (Oplatka 2011, p. 60), including ways to appropriately and purposefully express emotions to students, compared to teachers of other departments/disciplines. Second, they play a dual role, as they are not only expected to teach pre-service teachers how to teach, but also to act as a role model for them (Lunenberg et al. 2007) by enacting appropriate professional practice. Thus, competence in appropriate emotion management appears to be of particular relevance for teacher educators, as it is perceived as contributing to the professional development of pre-service teachers.

A few empirical studies have investigated emotion display in higher education; however these studies were not focused specifically on teacher educators. Lunenberg et al. (2007) as well as Carillo and Baguley (2011) observed that teacher educators have been overlooked in empirical research. Our literature search, which did not find any studies focusing on pre-service teacher educators’ emotion display, confirmed this (for teacher educators’ emotions in general, see for example Day and Leitch 2001). Although some role differences can be expected between teacher educators and teachers in other disciplines at university, it is also logical to expect that they share significant commonalities, in particular, the broader context of teaching young adults at university.

## Emotion display in higher education classrooms

The influence of emotions on behaviour is well documented in the literature. However, individuals are not passive and “helpless” receivers of their emotions; they can regulate and manage their emotions. Research on emotions in educational contexts indicates that emotion regulation plays a crucial mediating function between the experienced emotion and the subsequent behavioural reaction (e.g. control-value theory, Pekrun 2006). Thus, across all educational contexts, including university, a teacher’s ability to regulate his or her emotions can be as significant to teaching and learning as the experienced emotions themselves are.

As aforementioned, the display of emotion is an integral dimension of emotion regulation (Gross 1998, 2002), sometimes labelled “emotion management”, within the overarching construct of “emotional intelligence” (Mortiboys 2005; Perry and Ball 2005). The higher education literature is ambivalent on the issue of appropriateness of expression and/or suppression of emotions. In an overview, Gates (2000) identified studies in which researchers have argued for an emotionally neutral learning environment at university in order to create an optimal learning space. Such studies point to the criticality of suppression of teacher emotions. Other studies, however, have emphasized the importance of both teacher and student emotions, and their authentic display in facilitating effective learning and teaching, and maintaining psychological well-being (Cranton and Carusetta 2004). But not all emotions are equally likely to be expressed openly. While positive emotions are more easily expressed openly, intense negative emotions such as anger or disappointment for example, are often hidden, as shown in Gates’s study (2000), in which higher education teachers reported masking such negative emotions, leading to their perception of being “role-players”, and producing a discrepancy between experienced emotions and expressed emotions. Rather than showing negative emotions, teachers reported using humour or other emotion-regulation strategies to mask their negative feelings. This type of role-play was perceived as an element of their professional identity. This finding corresponds with Constanti and Gibbs’s (2004) study, which showed teaching in higher education required ‘emotional labour’ from teachers. Emotional labour is a concept from sociological research (Hochschild 1983), referring to the need for employees to display specific emotions as part of their job to achieve certain job-related goals (e.g., customer satisfaction). According to Constanti and Gibbs (2004), university teachers must perform emotional labour, as economically students are perceived as customers, and thus teachers are obligated to treat them accordingly, which includes emotion display. For example, teachers should refrain from exhibiting frustration or anger as “employees” in the university workplace, or at least display such emotions in an accepted, norm-accordant manner.

With respect to discrete emotions, McPherson et al. (2003) investigated appropriate and inappropriate anger display in a higher-education setting from students’ perspectives. The results showed that very intense and aggressive forms of anger display, such as yelling at, criticizing, or threatening students, were regarded as inappropriate anger reactions, as students experienced these forms of anger display as violations of the expected social norms. On the contrary, if teachers discussed their anger with students and attempted to understand their perspectives, students considered the teacher reaction appropriate. The study also showed that students had very sensitive reactions to teachers’ anger before they had developed a relationship with the teacher. Displaying anger at the beginning of the relationship-building process, therefore, might be more harmful than the same display later in the process, when secure relationships have been established (see attachment theory; e.g., Riley 2011).

This result suggests that the perception of the appropriateness of emotion display can be context-dependent and contingent on varying levels of contextual factors. In addition, culture, including specific cultural emotional rules (for individuals, for individuals in a certain profession, etc.), plays a role in determining what is perceived as appropriate or inappropriate emotion display in society generally (Kitayama et al. 2004; Mesquita 2007; Suh et al. 1998) and within the teaching profession (Zembylas 2005).

In sum, not only has there been little research examining emotion expression or emotion suppression in higher-education settings but to date the research has produced inconclusive results. Furthermore, there appear to be no studies focused on teacher educators. Accordingly, the aims of the present study were twofold: (i) to investigate how teacher educators manage their feelings in the classroom; this included emotion expression and suppression, as well as (internal) emotion regulation strategies expected to impact on emotion and teacher well-being, and (ii) to examine teacher perceptions about the functions of emotion display in classroom learning and teaching.

## The present study

To shed more light on the emotional display of teacher educators, we conducted a study on the teacher–student relationship, and associated teacher emotions as they arise during teaching at university. The study was undertaken at two public universities in Australia, and 15 teachers instructing students majoring in education participated in the study.

Two rounds of in-depth interviews were conducted, with 15 teacher educators (6 male, 9 female) participating in the first interview round. Of this 15, 9 (4 male, 5 female) also took part in the second round of interviews. The first interview focused on teacher emotions and emotion display when teaching and interacting with students generally (example interview question: “Do you show and express your feelings while teaching and interacting with students or do you also hide them sometimes? Could you explain?” followed by probes). The second interviews focused on the descriptions of concrete situations, as emotionally experienced by the teachers while teaching and interacting with their current first-year cohort. The focus on the current group of first-year students was the reason for the decrease in the number of participating teachers (from 15 in the first round to 9 in the second), as not all of the teachers were teaching first-year when the second round took place. The participants represented a cross-section of subject areas taught in teacher education (e.g., maths education, literacy education, science education, curriculum planning). Permission from the universities’ ethics committees, and written individual consent were obtained. Each interview lasted between 35 and 75 min and was digitally recorded for later verbatim transcription.

A phenomenological approach searching for overall themes and using codes to structure the interview material was adopted for the analysis of the interview data. The analytical process consisted of two phases:

*Phase 1* In the initial phase, the transcripts were read several times, and all text passages that could be allocated to the category of emotion display were electronically coded using the MAXQDA software with the descriptive code of “emotion display”. In addition, teachers spoke of emotion-regulation strategies that affected the internal regulation of their emotions, in terms of duration and intensity. These strategies are of importance to emotion display as well, as the successful regulation of experienced feelings in terms of duration and intensity (e.g., down-regulating anger) impacts how emotions might be displayed, or how successful an emotional suppression might be. Consequently, text passages referring to emotion-regulation strategies were additionally coded with the descriptive code “emotion regulation”.

*Phase 2* In the second phase, the coding was refined to distinguish between the perceptions of teachers with respect to *how to display positive emotions* and *how to display negative emotions*. Most responses were assigned to the dimension concerning how to express or hide negative feelings. The category of emotion-regulation strategies was also further differentiated into the following sub-categories: *cognitive strategies*, either (a) reappraisal or (b) acceptance of the situation by adapting expectations (Sutton 2004); *sharing feelings with others* (Sutton et al. 2009); and *establishing an emotional border* (“don’t put their monkey on my back”). Statements that referred to an overall lack of emotion regulation were allocated to the sub-category of *emotion suppression/no regulation*. The coding scheme is illustrated in Table 1.

**Table 1:** The applied coding scheme

Emotion display	This category addresses how teachers display emotions in the classroom, including their opinions about emotion display
Displaying positive emotions	This code is used when teachers talk about how to display positive emotions.
	Example: So, okay, the positive ones are easy to handle. Just join in, just share the fun.
Displaying negative emotions	This code is used when teachers talk about how to display negative emotions.
	Example: Probably. I am sure I do. I am a bit of an open book. So, I think, you know, I don’t… I don’t hide my feelings or even though I try to… As I’ve said I am not gonna show that I am angry.
Emotion-regulation strategies	This code addresses how teachers try to control and/or regulate their own emotions.
Cognitive strategy 1: reappraisal	The teacher tries to explain the situation (reappraisal), which helps to regulate the emotion.
	Example: But if somebody … dislikes me it doesn’t worry me, because I am… doing a role.
Cognitive strategy 2: acceptance by adapting expectations	The teacher reports setting realistic expectations, or he/she downgrades expectations in order to avoid negative emotions. He/she accepts the situation.
	Example: Yeah, it’s just fine. Yeah, yeah, that, yeah… I think you need to be very light-hearted about the position that you hold. It’s… it’s… It’s not the end of the world. So, yeah…
Sharing feelings with others	The teacher reports that sharing his/her emotions facilitates regulation.
	Example: Then I might, you know, talk to another… It’s quite isolated here. […] And … and we sort of chat. So I’ll say: Well, this really annoys me.
Establishing an emotional border	The teacher reports not feeling much empathy for students in terms of maintaining his/her own emotional balance.
	Example: Here… Deliver. Engage. Don’t have to put your monkey on my back.
Emotion suppression (no regulation)	The teacher reports that one must suppress one’s emotions (in particular, negative emotions) and then move on. No explicit statement about any particular emotion-regulation strategy is made.
	Example: I: And how do you normally handle these emotions? T: Move on.

## Findings

The results of the analyses are presented in three sections. We commence by reporting teacher educators’ views and reflections on positive emotions display when teaching and interacting with first-year pre-service teachers, followed by their views and reflections on negative emotions display. The third section presents the range of emotion-regulation strategies reported by teachers, and their reflections on how these strategies helped them internally regulate the intensity and duration of their emotional experiences, particularly the negative ones.

### Display of positive emotions

More than half of the participants reflected on the display of their positive emotions. All agreed that positive emotions (e.g., happiness, joy, enthusiasm, or humour) can and should be expressed openly in the classroom and in one-on-one settings with students. However, the interviews also revealed that the manner of expression depended on the teachers and their personalities, including their relational attitudes. Most reported that they expressed their positive feelings verbally by addressing their positive emotion (e.g., “I am really excited that…”), by praising students (“I am proud that …”), and by expressing humour (e.g., laughing about a joke) or by showing enthusiasm for or excitement about a subject. Although these accounts primarily addressed positive emotion display on a verbal level, one teacher, who characterized herself as a very relational-oriented teacher, reported a behavioural emotional reaction, namely hugging students, as shown in the following interview excerpt:

I would equally say, “I am so happy for you.” You know, if somebody gets a job or if somebody gets an award or something else. “So I feel so happy for you. It’s fantastic.” And you know, I would hug students and students would hug me. Not all the time but I wouldn’t hold back from doing that kind of thing. (I4/1, female)

Interestingly, another female teacher emphasized the opposite view, i.e., that relationships between students and teachers in higher education settings should be perceived as professional working relationships. Based on her experience this excluded emotion display, although this may not have been a conscious decision. Her elaboration statements indicate that she maintained a high emotional boundary in her interactions with students, which made it less likely that she would express her positive feelings with a hug as the previously quoted teacher did:

But I think I do the job as a real person. So you form relationships… on a level… a professional level with people […] And I don’t think I consciously don’t take that emotional part. I think that’s just the way I am. I don’t really… So, I don’t think it’s a conscious thing of I decide to use, you know, to make an emotional choice or not. (I5/1, female)

Another female teacher, who stressed the importance of avoiding overly intense emotions, both negative and positive, in particular when they are directed towards students, addressed the issue of maintaining stricter emotional boundaries in passing:

I get very enthused about the science or you know that type of thing. It’s really cool stuff. But as far as reacting to students, I try not to be up… way up or way down (I13/1, female).

In sum, the evidence presented here reveals that the display of positive emotions appeared to be unproblematic from the interviewees perspectives, at least as long as the display did not include overly intense emotional reactions, in particular behavioural demonstrations. However, the data also suggests that the personality of a teacher informs the emotional boundaries that he or she sets for him/herself. Some variation was detected between interviewees in terms of the perceived-as-appropriate as well as the utilized expression of positive emotions.

### Display of negative emotions

All participants perceived the control of negative emotions (which either entailed a total expressive suppression of the negative emotion or an as-appropriate-perceived display of that emotion) as a part of the professional behaviour expected of teachers. The perception of what an “appropriate” expression involved varied among these teachers, as evident in discussion of positive emotions. However, there was unanimous agreement that negative emotions, particularly anger, must be controlled and not displayed in an inappropriate manner from a teachers’ perspective (e.g., yelling or shouting at students; storming out of the classroom in anger). Although gender differences in this regard have been addressed in the literature (e.g., men being more willing to express anger than women; e.g., Timmers et al. 1998), in the present study no gender difference could be found in terms of managing negative emotions. Both female and male teachers expressed the same opinion regarding the necessity of the as-appropriate-perceived display of negative emotions. In addition, views of what was perceived as “appropriate” did not appear gender-specific, but rather individual-specific.

Some teachers reported endeavouring to suppress their negative feelings in the classroom (“put emotions on hold”; I10/1, female) to enable successful learning and teaching. One male teacher (I1/1) emphasized the importance of “putting personal feelings aside” for both students and teachers in order to promote effective learning and teaching. Another male teacher reported that putting negative feelings aside was important for his role as facilitator within the context of a positive student–teacher relationship. This teacher spoke about a one-on-one interaction with a student that he experienced as difficult. In this interaction, he suppressed his true feelings, as he described in the following statement:

And I just bit my tongue and I let him know that he needs to be more open-minded. […] You know that was probably one of those instances where you… if it was outside of school I probably would have said, you know: “Chill out! Or: What’s your problem?” You know, because these people just drive you wild like just there is more than one person in this world. The world doesn’t revolve around you. You know. But I didn’t say that. So that was just an example of sometimes people will annoy you, but you just have the smiley face and you go on with things, because it’s my role. So a person that is a facilitator. And if I have conflicts or friction, it’s going to be very difficult. So I try and leave that out of it. (I12/2, male)

Although most agreed teachers’ feelings should not interfere with classroom learning, many also reported that they were not willing to ignore negative incidents when they occurred, preferring to talk calmly on a one-to-one basis with particular students outside the classroom about such negatively experienced situations. In the educational literature, this approach is referred to as “assertive teaching”, which includes responses whereby “assertive teachers calmly, firmly, and clearly communicate to students their expectations for appropriate behavior” (McPherson et al. 2003, p. 87). The statement below illustrates this method of talking to students on a one-to-one basis. It also reveals the beneficial effect that taking time can have in regulating emotions. This example represents a combination of active-constructive (talking with the person you are angry with) and passive-constructive (using time to cool down) strategies of anger regulation (Rivers et al. 2007):

I think, you know, if you are really angry, I think you can say it. You can say, for example, you know, I wouldn’t say, whatever, you know: “You are an idiot.” All right? Or something like that. But I might say something like: “You know, your lack of professionalism at the moment makes me very angry and I’d like to have a word with you after class.” Or something like that. So I wouldn’t like to play it out in class. But I’d try to have a conversation. And that actually worked quite well. […] And it gives me time to come down as well (I2/1, female).

One male teacher touched on this aspect of emotion control, also commenting that losing control would lead to negative emotional reactions, such as embarrassment, which has been shown to be linked to the perception of self-worth (Parrott and Harré 1996; Robbins and Parlavecchio 2006):

And we just talk about it in a normal sort of kind of voice. Not get angry about it. […] I mean, if you really thumped the table and shared that stuff with a group of adults. You’d look a bit silly. (I9/1, male)

However, suppressing negative feelings or communicating them in an acceptable fashion appeared to require “acting behaviour” from teachers to some extent. One female teacher reported an incident with a student who asked many questions in class in an aggressive and challenging manner, which made it hard for her to control her feelings:

And then she would arrive. And I’d smile at her and knowing that my smile was completely insincere. And I was thinking: Oh no! Wish she hadn’t come. And she must have felt it, because I felt the antagonism. I am not that good an actress. And so on the surface I was trying to be the cool lecturer listening, dealing with something at a relatively superficial level, while underneath my emotions were very, very negative. What I wanted to do was pushing her over my knees and smacking her bottom. (I3/1, female)

Other accounts revealed feelings that the total suppression of experienced emotions could be counterproductive, as the following interview excerpt illustrates:

Because, you know, if I sort of pretend that everything is, you know, just fine. But in fact I am sort of, you know, boiling under the surface. That’s not good. Students feel it.

Q: Mhm, okay. So, you say, that it is important to show them [the emotions] whenever it is possible?

Yeah. I mean not in an abusive manner or aggressive. But, you know, you make it personal. You say, you know: “I feel really bad about this, you know, if you do such and such.” I think that I always try to be authentic. I don’t try to play a role. What I mean by that is: If, for example, something annoys me. Uhm, I’ll tell them. You know, because they would notice anyway. (I2/1, female)

Although authenticity appeared to be valued by many of the teachers, it also appeared that feelings deemed incompatible with the teachers’ sense of professionalism had to be suppressed. This caused tensions as teachers had to master the balance between displaying authenticity and simultaneously suppressing their negative emotions. Consequently, personal preferences (liking or disliking specific students) that could be accompanied by either positive or negative emotions had to be hidden since unacceptable from a professional perspective, as some accounts revealed. These teachers were unanimous that personal preferences should ideally be completely hidden in a teaching and learning setting:

So, and anyway, and then she… so she was in the office then… and she started chatting (annoyed voice), you know, about her life and yeah […] But then (sighs), you know, she is not my favourite student (laughs slightly; lowers her voice). So you know, it was, you know, we were getting on. I was doing all the right things. But you know, after a while I thought, okay, you know, can we leave this now. (I6/2, female)

Also, from teachers’ perspectives, emotions triggered by out-of-class circumstances (e.g., family, friends, other students at the university, colleagues) needed to be hidden for reasons of professionalism, particularly if negative in nature (e.g., sorrow due to relationship problems).

In addition to providing information on how teachers sought to maintain an effective learning environment and positive student–teacher relationships, the data also revealed another function of emotion-display control. When probed, all teachers interviewed addressed the special role they had within the general population of university teachers. More specifically, they saw their role as educating future teachers, and consequently perceived the need to serve as role-models, not only in terms of teaching but also in terms of managing and displaying emotions in professional practice. All participants were mindful of this role-model dimension of their work, as evident in the interviews:

That’s my home face. I might go home and get blablablabla (imitates expressing emotions, laughs). I don’t do it here [showing the emotions at university]. You know, you have to put a professional face on. […] You don’t have time to be emotional and think with your emotions. Even though you might be, you know, ticked off or annoyed, it’s not about your annoyance. Ultimately, we’re producing teachers to go out to schools and it’s about those kids in their classroom. So, you have to keep the big picture in mind. (I11/1, female)

Overall, participants also reported believing that negative emotions should be displayed in a norm-accordant way whenever possible, to maintain the highest possible degree of authenticity, and to achieve their educational goals (e.g., maintaining an effective classroom environment). Accordingly, many reported that an acceptable emotion display was frequently accompanied by a down-regulation of the emotion in terms of intensity or aggression (e.g., talking calmly with students when feeling angry, instead of shouting at them). However, the majority also reported that some situations required a complete masking of emotions, in particular if these emotions were perceived as incompatible with teacher professionalism (e.g., having personal preferences) or if the emotions were triggered by circumstances outside the classroom setting (e.g., stress with one’s partner).

### Applied strategies for the internal regulation of emotions

Many participants mentioned incidents that included descriptions of emotion-regulation strategies that facilitated the regulation of their internal experience of emotions (in terms of occurrence, intensity, or duration).

Some reported strategies that fit Gross’s (1998) description of “response-focused” strategies, which deal with the regulation of already-generated emotions. A strategy frequently mentioned was sharing one’s emotions, positive and negative, with significant others, for example, members of one’s family or colleagues in the department. However, although one teacher highlighted the helpfulness of sharing negative emotions and discussing the incidents that led to these emotions, she also reported that there were few opportunities provided at the university to discuss such emotionally draining incidents:

Q: Okay. And if you feel angry with the students, do you show the anger then?

Oh, then I will. Then I might, you know, talk to another… It’s quite isolated here. You can see how it is set up even with the rooms. So a lot of the time it’s very isolated and it’s quite hard to sort of meet people sometimes. But you know, I’ve made a new friend last year, which is good (laughs). And we sort of chat. So I’ll say: Well, this really annoys me. Or you talk about some students and you find the others find the same as well. And this is a bit of co-comforting. And you then, yeah, you sort of talk about what you do. (I6/1, female)

Other response-focused emotion-regulation strategies revealed in the data were cognitive strategies: rationalization or acceptance of the situation by adaptation of expectations. In terms of rationalization, many teachers used rational arguments to explain difficult situations, which helped them to down-regulate their negative emotions. For example, one teacher reported that “giggly” girls sometimes annoyed him, but he rationalized the situation:

They are still in that kind of school-girl, school-boy mode, which is pretty normal at this… this stage. Halfway through first semester. […]I think I try and understand where they are coming from and not be too… faced by the fact that they are 17-year-old giggly girls at times. (I7/2, male)

In addition, acceptance of a situation by adapting one’s expectations also appeared to help prevent teachers from experiencing overly intense negative emotions, in particular those stemming from experiences of disappointment or frustration:

I accept some… some, boundaries… you know… some limits. I am a human, as you mentioned earlier, and I am… there are limits to what I can do. And I try hard but if, you know, I accept that you cannot succeed every time. (I14/1, male)

A few teachers reported using strategies that circumvented the occurrence of negative feelings, what Gross (1998) called antecedent-focused strategies. Strategies involved attempts to distance themselves from the emotional issues of students, a form of creating emotional boundaries. One teacher called this strategy “*not taking their monkey on my back*” (I15/1, male). However, the ability to maintain an emotional distance from students’ problems appears to be a skill that comes with experience, as the following example illustrates:

I think I’ve probably had enough experience of tears now. From boys and girls. … Goodness. I never say things like: Please, don’t cry. Okay? In fact I acknowledge their feelings without acknowledging the fact that they’re sobbing their hearts out. (….) I will always keep just that… just that bit of distance. It’s about not get… I suppose it’s what nurses do. It’s just about caring but not taking it to heart. Not actually getting completely… involved. Because you can’t. Really. You can’t. (pauses). Yeah, there is concern without taking on their worries. There is no point in me bursting into tears as well. (I3/2, female)

But not all teachers reported internal emotion-regulation strategies related to regulating the occurrence, intensity, or frequency of their emotions. Without any regulation, some tried to suppress their emotions in order to “function” within the profession, as briefly discussed in the section on the display of negative emotions. Most of the time, intense negative emotions—which had to be suppressed according to teachers—emerged not from the classroom, but were triggered by out-of-classroom factors apparently inherent to the profession (e.g., high pressure, high workload). Due to their conceptions of professionalism, these teacher educators maintained that such negative emotions ought to be hidden from students and colleagues in daily interactions, to the extent possible. But teachers were not always successful in the continuous suppression of negative emotions as the following quote illustrates:

But I don’t really talk about it [the high work-load]. The only time I’ve spoken about it, and this is not really good. It’s not in class. It’s a couple of times this year when my marking load is just… […] … but where I had so much marking to do. And then my emotions have come out. Because I’ve actually felt that I haven’t been able to cope. So, a couple of times I have actually had to cope with my… contact my boss and say: Look, I don’t know what I am gonna do, because I just cannot cope with my workload. I am working seven hours a day. You know, seven days a week. And then I get in at four o’clock in the morning and trying to mark for weeks and then I am going and try to teach them and I am just exhausted. And then… Then these emotions have come out. (I6/1; female)

In this section we have shown that the teachers interviewed employed a variety of emotion-regulation strategies that primarily functioned to help them cope with negative emotional experiences. These strategies were applied either before the emotion occurred (e.g., by setting emotional boundaries) or as a method of down-regulating an already-evoked emotion (e.g., by the reappraisal of a situation or by talking about negative emotions). However, although most of the teachers interviewed appeared to be competent in their use of emotion-regulation strategies, some admitted lacking experience in adaptive regulation, particularly when faced with difficult situations generated by the demanding work of academics in general. This potentially increases the likelihood of general, unregulated emotional suppression, and the associated risk of emotional overload.

## Discussion

The present study investigated teacher educators’ perceptions of appropriate and inappropriate emotion display by teachers during teaching and in interactions with students, as well as the functions of emotion regulation in practice. In the following section we discuss the findings of the study in relation to three bodies of literature: first, in relation to psychological research on emotion regulation, including a general psychological (e.g., Gross 1998) and a social-psychological perspective (e.g., Tamir 2011); second, in relation to applied (educational) research on teacher emotion-regulation in higher education, and occasionally in the school context; and third, in regard to the extant literature on the dual role of teacher educators as both teacher and role-model.

Beginning with the display of positive emotions, the results suggest that the teacher educators interviewed in this study viewed the open expression of positive emotions (such as fun, humour, or happiness) as an important aspect of teaching practice. However, this mode of expression appeared to be informed by the emotional boundaries that the respective teachers set for themselves (e.g., teachers had different perceptions of how much and what forms of emotion display were within accepted boundaries; see also Hayward and Tuckey 2011).

In terms of negative emotions, the need to control such experiences and occasionally also the need to completely conceal them, emerged from the data. Similar results were reported in school research (e.g., Aultmann et al. 2009; Liljestrom et al. 2007; Sutton 2004, 2007; Sutton et al. 2009). These findings might partly be attributed to the fact that many teacher educators were school teachers themselves before university teaching (Carillo and Baguley 2011). It is reasonable to assume that these teachers would have developed a form of habitus in regulating their emotions in teaching settings through their school teaching experience. Another explanation may be the similarities that the teaching profession generally shares across different educational contexts, suggesting that university teaching and school teaching may evoke similar emotional (display) reactions from teachers. For example, Thomas (2003) found that higher education teachers in nursing education displayed the same reactions as school teachers and teacher educators when experiencing anger: they were found to hide their anger, and on no account would express anger in an abusive way.

The findings of the present study indicate that emotion display in general, and control of negative emotions in particular, is inextricably linked to teacher educators’ perceptions of professionalism. Beliefs about professionalism are, in turn, influenced by various factors, such as social mores and rules regarding emotions, norms within the teaching workplace, or individual personality (Fischer et al. 2004), as well as prior teaching experience. The adage, “teachers teach as they are taught” (Lunenberg et al. 2007, p. 586) has been confirmed in numerous studies on teacher education. Teachers’ beliefs about teaching are seldom questioned, unless teachers engage in a conscious process of reflection.

In contrast to the university teachers in Constanti and Gibbs’ (2004) study, who emphasized the importance of suppressing negative emotions due to economic reasons (“students as customers have to be satisfied”), the findings of the present study highlight moral motivations inherent to the teaching profession (e.g., care for students) as the determinants of teachers’ methods of emotion display. Thus, in line with previous research on the morality of the teaching profession (e.g. Oser et al. 1992; de Ruyter and Kole 2010), our results also highlight the moral dimension of teaching in teacher education, and its impact on teaching practice. Similarly to Sutton’s (2004) interview study of American high-school teachers, the university teachers in the present study viewed the failure to control the display of negative emotions as a violation of their moral duty to facilitate learning and be role models for appropriate teaching behaviour. Further, that this type of failure would lead to negative emotional reactions, such as shame and/or embarrassment.

However, teachers’ capacity to hide particular emotions is variable, and some participants in this study reported that their emotions occasionally “came through”. Similar results were found in laboratory studies of emotion-regulation: participants in such experiments were able to control their emotional behaviour, but it was far more difficult to control nonverbal signs such as facial expressions (Butler and Gross 2004).

Moreover, the present study found that masking, or hiding emotions need not be negative or maladaptive in nature, as sometimes claimed in studies highlighting the burden of “emotional labour” that teachers must bear (e.g., Constanti and Gibbs 2004). Our finding is in line with the psychological framework of emotion regulation. According to Butler and Gross (2004), the adaptive or maladaptive nature of the strategy of the expressive suppression of emotions depends on the (social) context, as the expressive suppression of emotions might fulfil desired social functions, “benefiting the regulator, the social partner or their relationship” (p. 102) in the short and/or long term. Similarly, Hargreaves (2000) argues, “Yet, at its best, emotional labor in teaching (and in other occupations) can be pleasurable and rewarding—when people are able to pursue their own purposes through it” (p. 814). In the teaching context, although suppression may be experienced as emotionally exhausting by teachers in some situations, hiding emotions may also serve various beneficial functions, depending on the teacher’s short-term, long-term, intrapersonal and interpersonal goals, (Tamir 2011).

The teachers in our study addressed some of these instrumental functions of the conscious use of emotion display and/or emotion suppression. Firstly, according to the teachers interviewed, the communication of emotions or their masking in particular situations helped them maintain a productive and effective learning and teaching environment. Secondly, they reported that it supported the construction and maintenance of positive relationships between teachers and students. Thirdly, in terms of the role-model aspect of the job of teacher educators, communication or hiding of emotions demonstrated desirable teaching behaviour for students who are future teachers. This finding parallels Tamir’s (2011) view that people not only follow hedonic goals when regulating their emotions, as was widely asserted in emotion-regulation research for many years (i.e., they seek to continue experienced positive feelings and to reduce or avoid negative ones), but that they also perceive emotion regulation as instrumental in the pursuit of multiple simultaneous goals. Depending on the cost-benefit calculation (e.g., cost: short-term decreased well-being; benefit: a long-term positive relationship and a relaxed classroom atmosphere), emotion suppression or masking can ultimately serve either an adaptive or a maladaptive function.

Furthermore, although the majority of the teachers in this study agreed that masking true emotions might be beneficial in some situations, at the same time they emphasized the importance of adequate emotional expression for the benefit of authenticity in teaching (see also Cranton and Carusetta 2004; Kitching 2009). This ambivalence regarding expression may, at least in part, explain the diverse understandings of emotion display at university level teaching. The data showed that on the one hand, teachers sought to achieve authenticity. On the other hand, they attempted to stay within the emotional boundaries associated with their profession as teachers, and that attempt included emotion control. These ostensibly contradictory aims, or conflicting approaches, could prove problematic if a teacher was unable to strike a balance between authenticity and emotion control. These contradicting aims could create ethical dilemmas (see Ehrich et al. 2011), requiring resolution by the teacher.

With respect to authenticity, prior studies have shown that even young children have already developed expectations about the emotional reactions of the people they interact with. If these reactions are not demonstrated as expected, it is likely that children will become puzzled (Butler and Gross 2004). Similarly, one teacher in this study observed that students would recognize if she completely suppressed her feelings, because they anticipated certain emotional and/or behavioural reactions from teachers in “critical”, classroom-norm-violating situations. As a result, most of the teachers preferred to express their feelings in a socially accepted, down-regulated, relatively neutral yet still authentic fashion, rather than suppressing them completely. According to Zhang and Zhu (2008), “authenticity primarily involves the genuine expression of positive feelings, [and] it might also include the spontaneous expression of negative emotions” (p. 117). Our findings showed that the teachers interviewed reported deliberate efforts to regulate their “expression of negative emotions” to fall within what they considered to be acceptable professional and social bounds. They considered this a strategy that both supported an authentic communication approach and sustained professional role-model behaviour.

Although, as discussed above, the conscious suppression of emotions may fulfil some beneficial (social) functions in particular social settings, the total suppression of negative emotions without internal regulation can also be maladaptive in nature. Most of the teachers in our research applied various emotion-regulation strategies that could be categorized according to Gross’s (1998) distinction between antecedent-focused and response-focused strategies. These strategies helped them to regulate the internal experiences of their emotions, primarily in terms of duration and intensity, e.g. using rationalization in order to down-regulate negative emotional experiences. However, in some accounts, it was clear that regulation strategies were inadequate, and an overall internal and external suppression of negative emotions was reported. Emotion-regulation research (e.g., Gross 2002) has revealed that suppressing emotions without regulation is likely to intensify the internal experience of the respective negative emotion. There was evidence of this in the present study, as some teachers reported that they “boil underneath” if they try to completely suppress their emotions.

In sum, the findings suggest that total suppression of negative feelings together with non-regulation of such emotions, (and lack of opportunity for regulation, e.g., by sharing with colleagues or reflecting on and re-evaluating experiences) may *de facto* render being a teacher educator a profession demanding high emotional labour, imposing significant emotional burden. However, while school research revealed that emotional burden occurred most notably from teaching and interacting with students directly, the emotional burden of teacher educators often occurred by way of out-of-classroom factors within the profession. Specifically, teachers in school often expressed negative emotions when students were misbehaving or were having severe difficulties at home (Hargreaves 2005; O’Connor 2008; Riley 2011). By contrast, teacher educators frequently mentioned emotional burden triggered by high workload in marking, high pressure to publish or demand to complete a PhD to enhance their academic qualification and maximise promotional opportunity (e.g., Carillo and Baguley 2011; Ducharme 1993). Thus, university teacher educators appeared to experience relatively low emotional burden when teaching and interacting with students, but high competence in managing emotions in classroom-settings and direct interactions with students. From teacher educators’ perspective, “appropriate” ways of emotion display was a useful and irrefutable tool to facilitate the development of desirable classroom environments—cognitively, motivationally, emotionally, and socially.

## Conclusion

This study brings to light teacher educators’ reflections on teacher emotion display and its function, including several emotion-regulation strategies aimed at the regulation of internal emotional experiences. Although all teachers in this study reported competence in the regulation and display of their emotions, they stressed that a significant part of this competence was developed through experience. Thus, emotion-regulation strategies should be addressed in professional developmental courses for teacher educators, beginning in the early stages of the teaching career at university, which is frequently experienced as highly emotional (see Hagenauer and Volet 2013). Many—but not all—teacher educators have a background in school teaching and bring with them experience in emotion management in the classroom. Nevertheless it appears valuable to prepare teachers for effective emotional management, and role modelling for prospective teachers. Teaching expertise, including competence in emotion management, does not automatically emerge from experience, as experience is only one element of expertise. According to Fendler and Gläser-Zikuda (2013, p. 19) expertise can be described by three main factors: “specific abilities (i.e. competences), years of studying or practicing in a domain (i.e. experience), and knowledge (i.e. cognition).”

Furthermore, to develop expertise as teacher educators and role models, there may be benefits in modelling teaching strategies that demonstrate (purposeful) methods of emotion display in the classroom. This might involve encouraging teacher educators to describe strategies they use and why, linking theory and practice. The development of ability in using meta-commentaries in teacher education is highly relevant in light of evidence that without such commentaries, students often do not discern that their teacher educator is mirroring optimal teaching practice (Lunenberg et al. 2007, p. 590).

In addition, teacher educators who work with students from diverse cultural backgrounds or who come from another cultural background themselves may need to expand their knowledge and understanding of the diversity of culturally-appropriate emotion-display conventions and norms. Teachers and teacher educators need to be mindful that the perception of appropriate emotion display is culturally bound, including their own perceptions (Markus and Kitayama 1991).

In light of the increasing importance ascribed to excellence in university teaching as part of the discourse on “Scholarship in Teaching and Learning” (e.g., Kreber and Cranton 2000; Trigwell and Shale 2004), this applies to higher education teachers in general. All university teachers could potentially benefit from increased metacognitive knowledge of learning and teaching generally (Fendler and Gläser-Zikuda 2013), and the importance of emotions, emotion display and emotion regulation more specifically. The findings of this research suggest that the development of strategies for appropriate emotion regulation should be included in courses on effective university teaching.

Before addressing future research, the strengths of the present study, and two limitations related to the methodology used in this study, must be addressed.

In terms of strengths, the study connects psychological emotion regulation research (e.g. Richards and Gross 1999) with research on learning and instruction in university-based teacher education. While most of the psychological studies on emotion regulation were conducted in laboratory setting (e.g. using emotion induction), the present study was applied in the educational field, capturing the situated, context-bound nature of emotion regulation (Hagenauer and Volet 2013). Thus, the external validity of the findings was increased.

While studying the reflections of teacher educators on emotion display can be considered a strength of this research, the exclusive focus on this group reduces the generalizability of the findings to other university teachers. As aforementioned, teacher educators form a unique population of higher education teachers due to their prior teaching experience and dual role. This situation may have provided them more opportunities to reflect on the importance of emotion regulation. Furthermore, it is reasonable to expect that their university teaching practice is influenced by their prior school teaching experience. The interviews did indeed reveal that the participants in this study already possessed a relatively high competence in emotion regulation, according to the theoretical definitions of that cluster of competence (e.g., Gross 1998, 2002). University teachers from other disciplines may have different views on this topic and more varied levels of competence, as previous studies have shown that teaching approaches vary between disciplines (Lindblom-Ylänne et al. 2006). These approaches, in turn, are linked to teachers’ emotions (Trigwell 2012). Furthermore, as already stated, the present findings should not be generalized across different cultures, as emotion display is based on culture-specific rules (Boiger and Mesquita 2012; Markus and Kitayama 1991). Consequently, studies across different contexts are needed to allow more generalizable conclusions about emotion regulation, including emotion display in a variety of higher-education teaching and learning settings.

The data reported here relied on self-reports of university teachers and did not include observations, or students’ perspectives. Furthermore, the present data did not include information about teaching quality or learning success in classroom environments that may be linked to teachers’ display of emotions. These teachers’ views about the functionality of certain emotion-display strategies were inevitably subjective, and may have benefited from triangulation using indicators that take into account the social nature of teaching and learning, and address the interplay between teachers’ emotion display and students’ (emotional) reactions, and vice versa. Possible influential contextual (moderator) variables (such as teachers’ beliefs about professionalism, individual personalities, culture, etc., e.g., Mesquita 2007) should also be taken into consideration. The inclusion of multiple perspectives, taking into account emotions, emotion regulation, and emotion display of all actors involved (students and teachers), and addressing potential consequences of these strategies for classroom teaching and learning, should also be considered in future research. Finally, a combination of data-collection methods, including self-reports and other-reports, supplemented by objective observations in natural learning and teaching settings in higher-education classrooms may provide insights into the dynamics of emotion display and emotion regulation.
